# Spinocerebellar ataxia type 21 exists in the Chinese Han population

**DOI:** 10.1038/srep19897

**Published:** 2016-01-27

**Authors:** Sheng Zeng, Junsheng Zeng, Miao He, Xianfeng Zeng, Yao Zhou, Zhen Liu, Kun Xia, Qian Pan, Hong Jiang, Lu Shen, Xinxiang Yan, Beisha Tang, Junling Wang

**Affiliations:** 1 Department of Neurology, Xiangya Hospital, Central South University, Changsha, Hunan, P.R. China; 2 Key Laboratory of Hunan Province in Neurodegenerative Disorders, Changsha, Hunan, P.R. China; 3 State Key Laboratory of Medical Genetics, Changsha, Hunan, P.R. China

## Abstract

Recently, mutations in transmembrane protein 240 (*TMEM240*) were identified as the cause of spinocerebellar ataxia type 21 (SCA21) in several French families. Clinically, SCA21 is characterized as an early-onset, slowly progressive cerebellar syndrome typically associated with cognitive impairment. To date, molecular screening of SCA21 has not been reported among patients of other ethnic origins or in other areas. Here we used Sanger sequencing to detect mutations in exons of *TMEM240* in 340 unrelated probands with spinocerebellar ataxia for whom commonly known causative mutations have been excluded (96 probands of autosomal dominant spinocerebellar ataxia families and 244 patients with sporadic spinocerebellar ataxia). As a result, a *de novo* missense mutation (c.509C > T/p.P170L) was identified in one sporadic SCA patient. The condition manifested as early-onset (30 years old), slowly progressive cerebellar ataxia accompanied by mild early evidenced mental retardation, mild frontal behavior disorders and intentional hand tremors. Although rare, a SCA21 case was identified and described in mainland China, thus broadening the ethnic distribution of SCA21 beyond French families.

Dominantly inherited spinocerebellar ataxias (SCAs) are a heterogeneous group of neurodegenerative disorders characterized by loss of coordination, progressive gait and limb ataxia^1,2^. To date, 34 different loci involving 27 genes have been reported for the SCA subtypes (http://neuromuscular.wustl.edu/ataxia/domatax.html). SCA21 was originally reported and described as a new subtype in a large French family in 2001^3^. Until recently, multiple missense mutations and a nonsense mutation in transmembrane protein 240 (*TMEM240*) were identified as the causality of spinocerebellar ataxia type 21 (SCA21, OMIM: #607454) in several French families including this original family^4^. Clinically, SCA21 is characterized as an early-onset, slowly progressive cerebellar syndrome typically associated with cognitive impairment. To date, molecular screening of SCA21 has not been reported among patients of other ethnic origins or in other areas.

Here, we used Sanger sequencing to detect mutations in exons and intron-exon boundaries of *TMEM240*. The purpose of this study was to investigate the genetic and clinical features of SCA21 in patients from mainland China.

## Results

### Result of mutation detection

**Figure 1:**
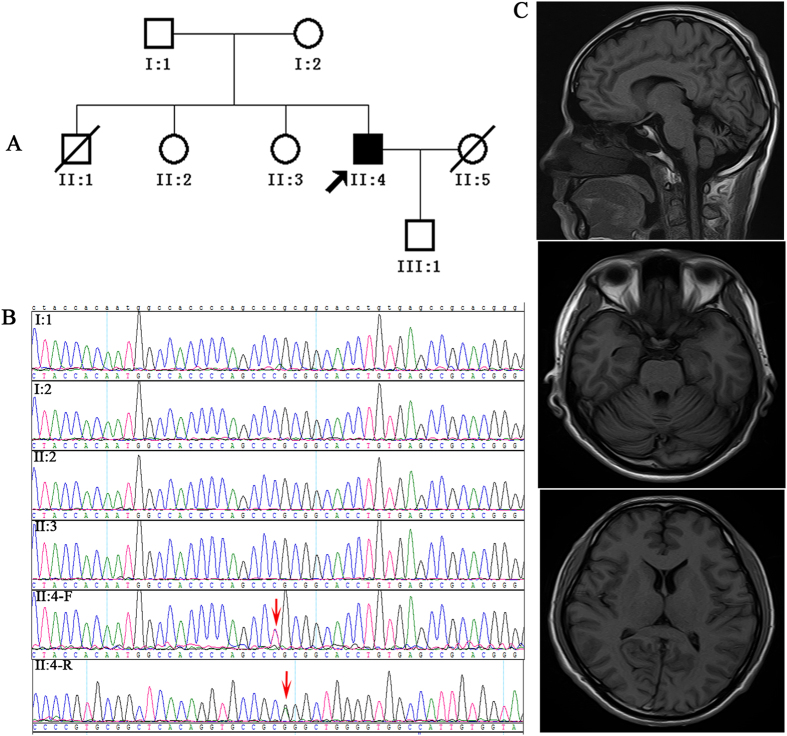
Clinical and genetic data of the patient with SCA21. (**A**) Patient pedigree (Arrow points to affected patient); (**B**) Electropherogram of Sanger sequencing of PCR products from the members of the patient pedigree. From top to bottom: I:1 (father), I:2 (mother), II:2 (sister), II:3 (sister), II:4-F/II:4-R (proband, F and R represent forward and reverse sequencing, respectively). Red arrow indicates the mutation (c.509C > T). (**C**) Cranial magnetic resonance images: mild cerebellar atrophy was indicated.

A missense mutation (c.509C > T/p.P170L) in exon 4 of *TMEM240* was identified in one sporadic SCA patient (Fig. 1B). However, this mutation was not present in either of his parents or his other family members.

### Clinical features of the patients with a *TMEM240* mutation

The patient was a 40-year-old male from Hunan province of Mainland China. He had poor memory and mild mental retardation compared to his peers, which was observed early in his life at school. However, his motor skills were generally normal prior to the age of 33 years. At that time, his hands began to exhibit clumsiness in movement and tremors while holding objects. Soon, the patient developed an ataxic gait and clumsy walking, which slowly, but progressively, worsened. Slurred speech was also observed during the disease progression. The clinical examination revealed hyperreflexia in the knee, while no evidence of pyramidal signs and sensory loss was observed. Examinations of the cranial nerves were normal. Poor results on the finger–nose test, poor heel–kneel–shin ataxia and positivity for Romberg’s sign were also noted. The patient’s scores on the scales of motor and cognition listed above are summarized in Table 1. In general, the patient performed very poor on the motor and cognitive rating tests. In addition, the results of the NPI indicated the presence of frontal behavior disorders (delusion, aggressive, apathy, etc.).

His parents were 71 and 72 years of age and were normal, and no other individuals in the family, including his two elder sisters, were diagnosed with cerebellar ataxia. The missense mutation identified in this patient was not found in his family members (Fig. 1A).

The results of the routine blood test, liver function test, blood glucose test, blood lipid test, ceruloplasmin test and vitamin test were all within the normal ranges. No unusual findings were detected by electromyography, BAEP, VEP or SEP. MRI indicated atrophy of the cerebellum (Fig. 1C).

## Discussion

SCA21, a newly demonstrated SCA subtype, is an autosomal dominant neurologic disorder characterized by onset in early life of slowly progressive cerebellar ataxia, which is associated with cognitive impairment in most patients^4^. Five causative missense mutations and one truncating mutation were identified in the *TMEM240* gene in affected members of eight unrelated French families, among whom three families shared the same original mutation (c.509C > T/p.P170L).

In this study, a large number of Chinese patients with SCA were screened for the SCA21 mutation, and a *de novo* missense mutation (c.509C > T/p.P170L) was identified in one sporadic SCA patient. In addition to two *de novo* mutations (c.509C > T/p.P170L and c.239C > T/p.T80M) found in two French families, this *de novo* mutation again indicates the presence of spontaneous events in this gene, suggesting that mutation c.509C > T/p.P170L may be a mutational hot spot. This phenomenon suggests that high regional mutation rates may exist in this telomeric region of chromosome 1. Regional mutation rates are subject to a variety of intrinsic characteristics and extrinsic factors^5^. Multiple factors (DNase hypersensitivity, high GC content, elder parental age, etc.) can explain the relatively high regional *de novo* mutation rates in this gene. To the best of our knowledge, this is the first report of a SCA21 subtype in the Chinese Han population and the first report of this disease in an ethnic group other than the French.

The main clinical features of this patient were early onset, slowly progressive cerebellar ataxia accompanied by mild early evidenced mental retardation, mild frontal behavior disorders and intentional hand tremors. The results of the laboratory tests, EMG, BAEP, VEP and SEP were all negative. There were no obvious differences in the clinical characteristics of our patient and those reported previously in French patients.

In conclusion, this is the first documentation of a Chinese patient with SCA21, which extends the ethnic association of this disease beyond families of French origin. Further clinical and genetic studies should be performed to shed light on the molecular mechanism underlying SCA21.

## Patients and Methods

### Subjects

Sanger sequencing was used to detect mutations in exons of *TMEM240* in 340 unrelated patients with spinocerebellar ataxia recruited from the outpatient neurology clinics of Xiangya Hospital of Central South University from February 1994 to December 2014. The clinical diagnosis of SCA was made based on the criteria proposed by Harding^1^. The 340 unrelated patients included 96 index patients with ADCA families and 244 index patients with S-SCA, in whom causative mutations responsible for SCA1/2/ 3/6/7/8/12/17/DRPLA and Friedreich ataxia had been excluded. Informed consent was obtained from all subjects, and the study protocol was approved by the Ethical Committee of Xiangya Hospital of the Central South University in China (equivalent to an Institutional Review Board) and carried out in accordance with the approved guidelines.

### Mutation detection

After obtaining informed consent, blood samples were obtained from the 340 patients described above. Genomic DNA was extracted from peripheral blood using standard extraction methods. Mutation analysis of the four coding exons and intron-exon boundaries in *TMEM240* was performed using PCR. The resulting amplicons, including the intron-exon boundaries, were screened for mutations via Sanger sequencing using an ABI 3700 instrument (Applied Biosystems). The primer sequences are shown in Table 2.

### Clinical investigation

For one positive subject, the clinical history was obtained and a clinical examination and evaluations, including the Scale for Assessment and Rating of Ataxia (SARA), International Cooperative Ataxia Rating Scale (ICARS), Wechsler Adult Intelligence Scale (WAIS), Raven’s Progressive Matrices (RPM), Montreal Cognitive Assessment (MoCA), Clock Drawing Task (CDT), Mini Mental State Examination (MMSE) and Neuropsychiatric Inventory (NPI), were performed. Systemic laboratory tests and diagnostic examinations were also performed to assess the clinical features objectively. The laboratory tests included a routine blood test, liver function test, blood glucose test, blood lipid test, ceruloplasmin test and vitamin test (vitamin A/vitamin B1/vitamin B2/vitamin B9/vitamin B12/vitamin C/vitamin D/vitamin E). The diagnostic evaluations included a neuroimaging study (MRI, scanning) and an electrophysiologic study (electromyogram/brainstem auditory evoked potential/visual evoked response/somatosensory evoked potential).

## Additional Information

**How to cite this article**: Zeng, S. *et al.* Spinocerebellar ataxia type 21 exists in the Chinese Han population. *Sci. Rep.* **6**, 19897; doi: 10.1038/srep19897 (2016).

## Figures and Tables

**Table 1:** The patient’s scores on scales of motor and cognition.

Rating scale	ICARS^a^	SARA	WAIS	RPM	MoCA	CDT	MMSE
Full score	100 (34,52,8,6)	40	/	60	30	4	30
Patient’s score	38 (9,25,4,0)	13	62	8	6	1	16

^a^Items of rating: posture and gait disturbances, kinetic functions, speech disorders and oculomotor disorders. /: rating as ranked distribution (≤69 is considered mental retardation).

**Table 2:** Primer sequences used in mutation analysis of *TMEM240* gene.

Primer ID		Sequence (5′–3′)
Primer 1	Forward	GGCGGACGATCCAGGGAA
	Reverse	GCACGGGCGGTAAACAAGG
Primer 2	Forward	CTGCGATCCCCATACCCG
	Reverse	CCCGCCCACCTTGAGACAG
Primer 3	Forward	CGCTGTCTGCCCGGACTTCA
	Reverse	GCCAGGTCCACGAGCCATCT
Primer 4	Forward	CGCCTCCGAGAACTACTTTGTG
	Reverse	GGGATGAGTCCGCCCTTGT
